# Clinicopathological features and the value of differential Cytokeratin 7 and 20 expression in resolving diagnostic dilemmas of ovarian involvement by colorectal adenocarcinoma and vice-versa

**DOI:** 10.1186/1746-1596-3-39

**Published:** 2008-09-18

**Authors:** Bharat Rekhi, Sophia George, Bhulaxmi Madur, RF Chinoy, Rajesh Dikshit, Amita Maheshwari

**Affiliations:** 1 Department of Pathology, Tata Memorial Hospital, Mumbai, India; 2 Department of Epidemiology and Biostatistics, Tata Memorial Hospital, Mumbai, India; 3 Department of Gynaecology, Surgical Oncology, Tata Memorial Hospital, Mumbai, India

## Abstract

The distinction between metastasis from a colorectal adenocarcinoma into the ovary and an ovarian adenocarcinoma is vital, but challenging at times, due to overlapping morphological features. Similarly, a distinction between an ovarian metastasis into the colorectum and a colorectal adenocarcinoma, although rare; is important and can be daunting. We report an analysis of 20 cases of ovarian involvement by metastatic colorectal adenocarcinomas and colorectal involvement by metastatic ovarian adenocarcinomas, including the value of differential expression of cytokeratins 7 & 20 by immunohistochemistry (IHC), in these cases. Nine cases (45%) were identified as colorectal adenocarcinomas metastatic to the ovary. On biopsy, all these cases showed a 'garland-like' tumor necrosis, with desmoplasia and predominantly exhibited a tubuloalveolar pattern (67% cases). On IHC, all 8 of 9 such cases, where staining for cytokeratin 20 was performed, displayed strong positivity and 7 cases, where staining for carcinoembryogenic antigen (CEA) was performed, revealed positivity for this marker (100%). Other 11 cases (55%) were ovarian adenocarcinomas, metastatic to the colorectum. These showed metachronous presentations, with the ovarian tumor preceding the colorectal tumor deposits. Morphologically, psammomatous calcification was noted in 73% of these cases, whereas 'garland-like' necrosis was absent in all. The chief morphological subtype was serous papillary cystadenocarcinoma (55% cases). On IHC, CK7 and CA 125 were positive in all 6 of 11 such cases, whereas CK 20 was negative in all these cases.

In cases of complex presentations like an ovarian involvement by a metastatic colorectal adenocarcinoma and vice-versa, certain clinicopathological features are useful. Differential expression of CK 7 and CK20 is vital in resolving these dilemmas. CK20 positivity and CK7 negativity is associated with a colorectal adenocarcinoma. Markers like CEA and CA-125 have an added value.

## Background

The ovary is a site for a wide range of tumors, both primary and metastatic. Approximately 10–30% of ovarian tumors are metastatic, commonly from the breast, stomach, and the colon [1]. Of these, colorectal metastasis account for approximately 4% [2]. Morphologically, these tumors mimic primary ovarian adenocarcinomas, especially ovarian endometrioid adenocarcinomas. Rarely, colorectum becomes a site for a metastasis from an ovarian adenocarcinoma [3]. Identification of the correct primary tumor in both the described presentations is necessary for an optimal management, including, specific chemotherapy (CT) in advanced stages. Whereas, ovarian adenocarcinomas respond to platinum based CT, cases of colonic adenocarcinomas are candidates for 5-fluorourocil based CT. [4]. The known clinical features favoring a metastatic tumor into the ovary include history of an antecedent malignancy, relatively smaller size of the tumor ~10 cms, solid structure, frequent bilaterality; absence of ascites and elevated serum carcinoembryonic antigen (CEA) levels [1]. Certain morphological features indicative of metastasis from colorectum include 'garland-like' tumor necrosis, segmental destruction of glands and absence of squamous metaplasia, whereas cribriform growth patterns and intraluminal "dirty" necrosis are indicators of endometrioid ovarian adenocarcinomas [5,6]. However, problem exists in sorting out these tumors, including in cases mucinous adenocarcinomas, when there is simultaneous involvement of the ovary and the colorectum at similar (synchronous) or at different (metachronous) times [6,7]. Lately, cytokeratins 7 and 20 have emerged to resolve these diagnostic dilemmas [7-9]. We sought to evaluate distinct clinicopathological features, along with value of differential expression of CK7 and CK20 in certain cases of ovarian involvement by metastatic colorectal adenocarcinomas, as well as in uncommon cases of colorectal involvement by metastatic ovarian adenocarcinomas.

## Methods

Twenty cases of ovarian tumors, including colorectal adenocarcinomas metastatic to the ovary and ovarian adenocarcinomas metastatic to the colorectum, were retrieved from the surgical pathology records, over a period of 5 years at a tertiary cancer referral centre. These included 'in-house' specimens as well as paraffin blocks and slides for review. Histologic confirmation of either ovarian or colorectal involvement was present, with radiological confirmation of the metastatic deposits. Cases of metastatic adenocarcinomas to the ovary from other sites were excluded.

Clinical details were retrieved from the charts and departmental electronic medical records (EMR). These included parameters like age, anatomic site of primary malignancy, radiological details with serum tumor marker levels, stage of the disease; disease status like synchronous or metachronous involvement and additional metastasis, if any.

Wherever available, gross features of the ovaries were tabulated including laterality, presence of capsular breach, surface component and presence of solid or cystic areas on cut sections. Haematoxylin and eosin sections (H&E) were available in all cases. Mucicarmine stain was employed in cases of mucinous or 'signet-ring' cell tumors.

Histomorphologically, the tumors were evaluated for type, degree of differentiation, presence of normal ovarian parenchyma, presence of 'garland-like' necrosis, defined as cystic glandular structures containing necrotic debris encircled by an array of round tubular glands [3]; lymphovascular emboli (LVI), stromal luteinization, desmoplastic response, squamoid differentiation, and psammomatous calcification.

Sections from the colorectal tumor were studied for tumor type, degree of differentiation and stage, including location of tumor i.e. whether mucosal or serosal, & invasion of the muscularis propria.

Immunohistochemistry (IHC) was performed on formalin fixed, paraffin embedded tissue sections by immunoperoxidase technique using the avidin-biotin method. The various antibody markers along with their respective dilutions, code and antigen retrieval method included cytokeratin (CK) 7 (1:100, OV-TL-12/30, DAKO, monoclonal, code M 7018; by microwave heating), CK 20 (1:50, Ks 20.8, DAKO, monoclonal, code M 7019), carcinoembryogenic antigen (CEA) (1:600, DAKO, polyclonal; by enzymatic i.e. pepsin digestion) and CA-125 (1:50, OC 125, DAKO, monoclonal, code M 3519; by microwave heating). The location of staining was graded as luminal, membranous or cytoplasmic. The intensity of staining was graded on a scale on 0–4 as: -Negative-<5%, positive->5% tumor cells positive. Semiquantitative scoring was as: 0 – < 5%, 1+ – 6%–25%, 2+ – 26%–50%, 3+ – 51%–75%, 4+ – 76%–100%. Focal staining was interpreted as positivity in ≤ 50% tumor cells and diffuse staining was interpreted as positivity in > 50%. [7]. Appropriate controls were included.

## Results

Out of 20 cases, 9 (45%) were of colorectal adenocarcinomas, metastatic to the ovary and 11 (55%) cases were of ovarian adenocarcinomas, metastatic to the colorectum.

### I) Cases of colorectal adenocarcinomas metastatic to the ovary

Among these cases, median age of presentation was 55 years. The presentation was synchronous in 6/9 (66.6%) cases. In view of most of these cases being referrals with limited details, the duration between the two lesions in cases with metachronous involvement could not be procured.

Out of 6 cases with available details on laterality, 4 (66.7%) were unilateral and 2 were bilateral (33.3%). On radiological and gross examination, both, solid and cystic pattern was the commonest, noted in 4/5 (80%) cases. Further, capsular breach was noted in all 3 specimens available for gross examination. The primary tumor was located in the sigmoid colorectum in 5 (83.3%) cases and in the ileocaecal region in 1 (16.6%) case. Colonic resection specimens were available in 2 cases; of which 1 was of stage T3 N0 Mx and the other was T4 N2 Mx at the time of diagnosis.

CEA levels were elevated preoperatively in 4 (90%) of 5 cases, where these were available.

#### Microscopic findings

Morphologically, tubuloglandular/endometrioid morphology was seen in 6 cases; 2 cases were of mucinous type, while one had 'signet-ring' cell morphology. 'Garland-like' necrosis and desmoplasia was noted in all 9 (100%) cases. Luteinization of the stroma and squamoid differentiation was not noted in any case. Lymphovascular emboli (LVI) were present in 5 (55.5%) cases. Psammomatous calcification was seen in 1 case.

The colonic resection or biopsy, received in 4 cases, showed mucosal dysplasia, confirming colonic origin of the primary.

**Figure 1:**
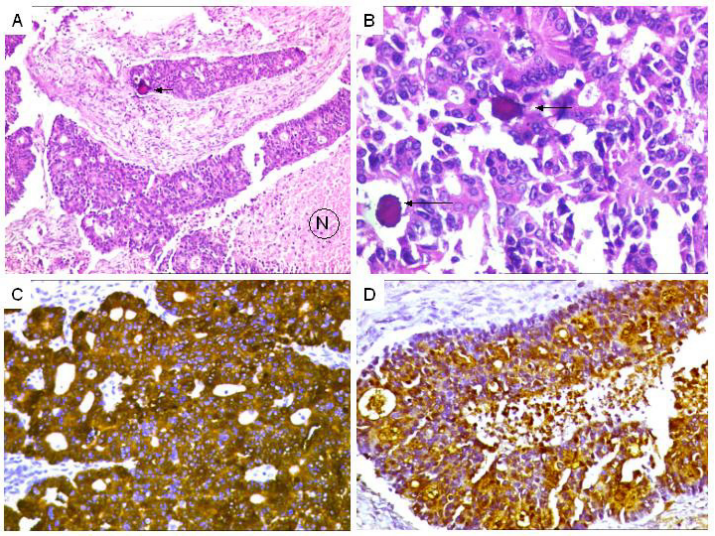
**A. Case of a metastatic colorectal adenocarcinoma to ovary, comprising areas of necrosis (N) surrounded by tumor cells. Focal psammomatous calcification (arrow).** H&E × 100. B. Higher magnification showing tumor cells displaying acinar arrangements with psammomatous calcification (arrows). H&E × 100. C. Diffuse CK 20 positivity. DAB × 400. D. Diffuse CEA expression. DAB × 200.

**Figure 2:**
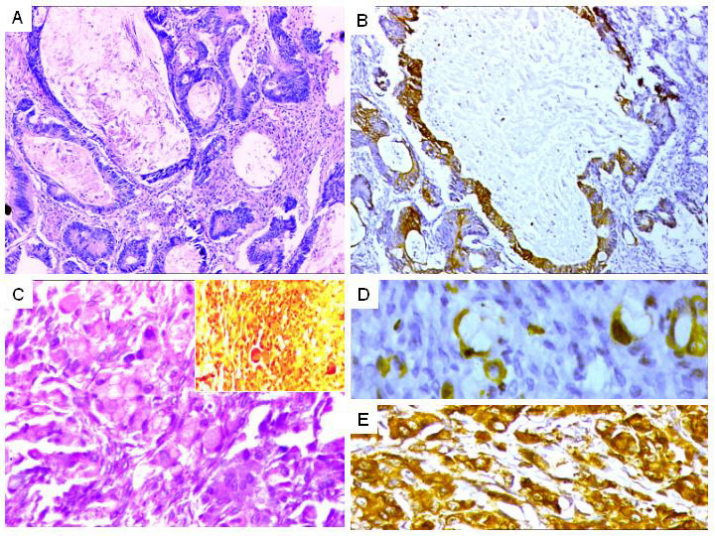
**A. Mucinous adenocarcinoma B. IHC results. Diffuse CK20 positivity and CK 7 negativity (not shown), reinforcing a metastatic colorectal adenocarcinoma.** C. Metastatic 'signet-ring' cell adenocarcinoma from colorectum. Infiltrating 'signet-ring' cells in a desmoplastic stroma. Inset, showing discrete mucicarmine positivity, highlighting intracytoplasmic mucin in the tumor cells. D. CK20 positivity in the signet-ring cells. E. Diffuse CEA expression. CK 7 was negative (not shown).

**Figure 3:**
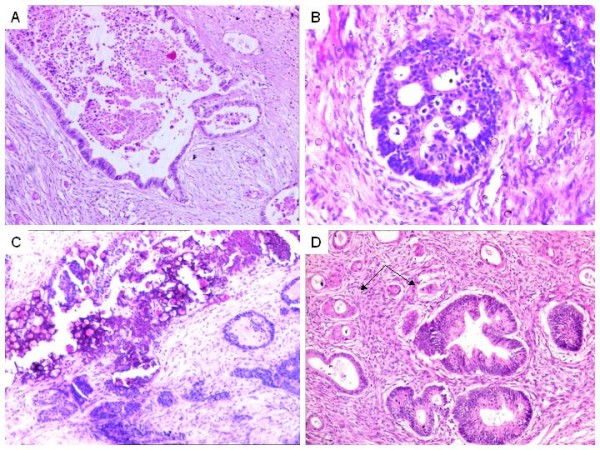
**Histomorphological patterns in colorectal adenocarcinomas.** A. 'Garland-like' necrosis in cases of metastatic colorectal adenocarcinomas. B. Intraluminal bridging with necrotic debris in cases of endometrioid type of primary ovarian adenocarcinoma. C. Conspicuous psammomatous calcification in cases of papillary serous cyst adenocarcinomas of the ovary. D. Squamous differentiation (arrow) in endometrioid adenocarcinoma of the ovary.

**Figure 4:**
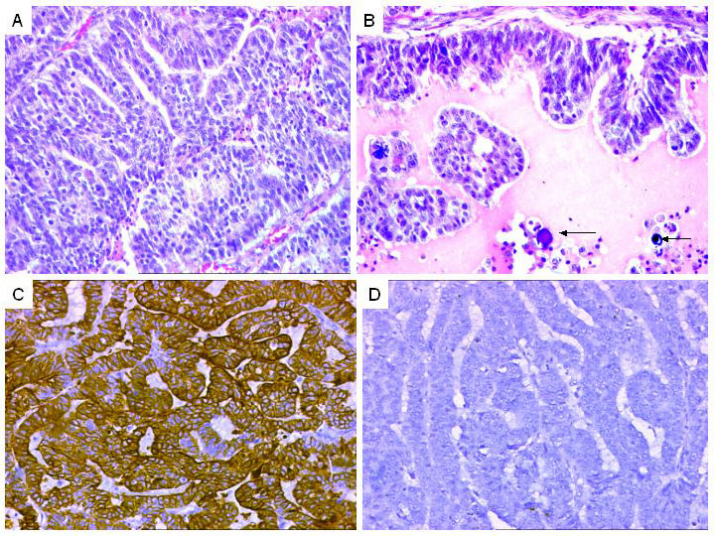
**A. Primary ovarian adenocarcinoma showing slit-like spaces reminiscent of endometrioid pattern. **H&E × 200. B. Focal psammomatous calcification (arrows). H&E × 200. C. IHC results. Positive CK 7 expression. DAB × 200 D. Negative CK20 expression. DAB × 200.

IHC was performed in 8 cases. All these cases displayed diffuse (4+) positivity for CK 20. CK7 expression was negative in 5 and focally positive (1+) in 3 cases. In addition, variable CEA positivity was observed in 7 cases, where it was performed. CA-125, performed in 4 cases, was negative. (Additional file 1) (Figures 1, 2, 3 &4A)

### II) Cases of ovarian adenocarcinomas metastatic to thecolorectum

Among these, the median age of presentation was 49 years. 81.8% cases were metachronous, with the ovarian primary preceding the colorectal involvement, while in one case the patient presented with the tumor of sigmoid colonic before the ovarian tumor was detected. The median duration between the colorectal and ovarian involvement was 3 years. Two cases had synchronous involvement of the ovaries with the colon.

Seven of 8 cases had bilateral ovarian involvement, with capsular breach in 5 of 6 cases, where gross details were available. Status of exact tumor location could be procured in 7 cases. Of these, the tumor was identified in the sigmoid colon and rectum in 4 (57.1%) cases, and in the proximal colon in 3 (42.8%) cases.

#### Microscopic findings

Morphologically, 6 cases were of serous papillary type, 2 of endometrioid type and remaining 3 cases were of poorly differentiated adenocarcinoma not otherwise specified (NOS).

Lymphovascular emboli were noted in 3/11 (27.2%) cases. Squamoid differentiation, stromal luteinization was absent, while psammomatous calcification was noted in 7/11 (63.6%) cases. Desmoplasia was noted in 1 case.

The tumor in the colorectum was predominantly submucosal, in the serosa or in the wall, without mucosal dysplasia in the 9 cases, including colonic resections or biopsies.

**Figure 5:**
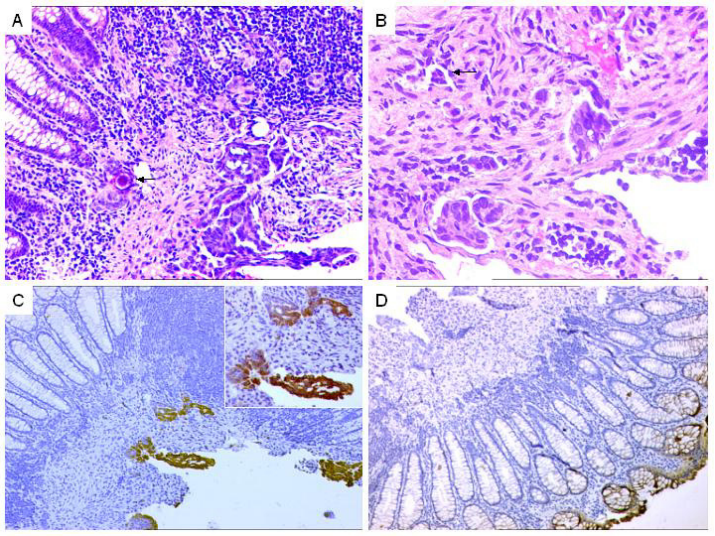
**Case of a papillary serouscystadenocarcinoma metastasizing to the sigmoid colon.** A. Clusters of tumor cells invading the lamina propria and the submucosa. Overlying colonic mucosa is normal. H&E × 200. B. Lymphovascular emboli in the submucosa. H&E × 400. C. IHC results. CK 7 highlighting the tumor cells. DAB × 100. Inset showing higher magnification of CK7 positive tumor cells. DAB × 200. D. Negative CK20. Superficial colonic mucosa showing positivity was an internal positive control. DAB × 100.

IHC was performed in 6 (54.5%) out of 11 cases. CK7 was diffusely positive in all 6(100%) cases, while CK 20 was negative in all cases. CA-125, performed in 3 cases, was positive (100%). CEA, performed in 2 cases, was negative. (Additional file 1). (Figures 4B, C, D &5).

## Discussion

The histomorphological features of ovarian and colorectal adenocarcinomas overlap, at times. In certain situations, the ovarian metastasis form a colorectal adenocarcinoma might be the primary presentation or a colorectal tumor might actually be a metastatic adenocarcinoma from the ovary [3,6]. Identification of an exact primary tumor type i.e. either ovarian or colorectal, in this scenario becomes vital for an appropriate management. Apart from certain clinicopathological features, lately, differential expression of cytokeratins 7 & 20 has been found to be useful in resolving these dilemmas [9-11]. The present study was based on clinicopathological features and identification of value of differential expression of CK 7 and 20 by immunohistochemistry, in cases of ovarian involvement by colorectal adenocarcinomas and. in cases of colorectal involvement by ovarian adenocarcinomas.

Among the already described clinical features, primary ovarian adenocarcinomas are usually know to occur at a relatively younger age group, as was seen in our study (median age = 49 years), in contrast to ovarian metastatic deposits from colorectal adenocarcinomas that were seen in an older age group (mean age = 55 years), as noted in an earlier study [1]. In our series, involvement of the ovary and colon in cases of colorectal metastasis was more commonly synchronous, in contrast to the findings of Lewis et al [11], wherein the ovarian lesions were identified prior to the colonic primary in 32% cases. This might be due to the fact that that ours being a referral centre, majority of the patients present at an advanced stage disease, along with existing lack of routine gynaecologic or gastrointestinal (GI) screening.

The differences in the pre and perioperative characteristics of primary and secondary ovarian tumors have been studied by Antila et al [1], who found that tumors that were relatively smaller -10 cms sized; solid in nature; presenting with ascites; in association with raised serum CEA & tumor-associated trypsin (TAT-1) inhibitor levels, were predominantly metastatic. Metastatic tumors are reported to be commonly bilateral [1,6,11]. However, we observed 66% of metastatic colorectal adenocarcinomas in the ovaries as unilateral masses, in as noted by Daya et al [12]. In their study, Lewis et al [11] have also reinforced this fact, with a suggestion of consideration of metastatic tumor in cases of younger patients with large sized tumors. Wherever the gross findings were available in our study, all such cases had a capsular breach and were both solid and cystic in maximum cases. The mean ovarian tumor size could not be ascertained in view of referral cases being submitted with limited details, adding to the diagnostic challenge. In addition, 90% of such cases displayed raised serum CEA levels, wherever available.

On histology, 'garland-like' necrosis with desmoplasia were the most valuable clues in diagnosing a metastatic colorectal carcinoma, as noted in earlier studies [2,5,6]. However, we did not observe stromal luteinization in any of our cases as noted by Mc Cluggage et al [6] in their cases. Metastatic mucinous adenocarcinomas can closely mimic primary ovarian, although, the latter, is invariably known to display areas resembling borderline primary ovarian neoplasms. However, this feature can be seen in a metastatic colorectal adenocarcinoma [11]. Both our cases of this subtype had histological, as well as gross evidence of surface involvement, with discrete features of a colonic adenocarcinoma, including 'garland-like' tumor necrosis. Presence of lymphovascular emboli favoured a metastatic carcinoma over a primary. Metastatic colorectal adenocarcinomas invariably mimic an endometrioid type of ovarian adenocarcinomas. Identification of foci of squamous differentiation, endometriosis or adenofibromatosis are pointers towards an ovarian adenocarcinoma. Despite these clinicopathological features, difficulty exists, wherein IHC is valuable in forming a more objective diagnosis [6].

Lately, cytokeratins 7 and 20 have been found to be useful in providing a direction towards exact primary in cases of metastasis with unknown primary (MUP) [10]. Cytokeratin 7 (CK7) is a basic (type II) cytokeratins, found in human ductal, glandular and transitional epithelia, while CK 20 it is normally expressed in gastrointestinal epithelium, urothelium and Merkel cells [13,14]. As described lately, these specific cytokeratins, with additional markers like CEA and CA125, forming an optimal IHC panel, have been documented to be helpful in resolving these dilemmatic situations of ovarian involvement by colorectal carcinoma and vice versa. The colonic tumors usually express CK 20 and CEA diffusely and are negative for CK7 and CA-125, the latter two being more frequently expressed by ovarian adenocarcinomas in this clinical context. This pattern of IHC expression helped in ascertaining metastatic colorectal adenocarcinoma in the ovary in 8/9 cases in our study. The remaining 1 case was spared with CK 20 expression since it had identical histomorphological of the colorectal adenocarcinomas that displayed a transition from the dysplastic colorectal mucosa. CK7 was focally positive in 3 such tumors, as noted earlier. [15] Moreover, CK 20 and CEA positivity can be observed in mucinous ovarian neoplasms. However, the expression is focal, unlike diffuse expression noted in lower GI adenocarcinomas [6,7,16]. In our limited number of cases in this clinical context, we did not find any pure mucinous ovarian tumor, although, focal mucinous differentiation was noted in a single case. Serum CEA levels were marginally elevated, but IHC for the same was negative. Lately, CDX2 and villin have been found to be 100% positive in cases of metastatic colorectal adenocarcinomas [11]. However, CDX2 can be seen in mucinous ovarian adenocarcinoma.

Eleven cases in our series were identified as primary ovarian adenocarcinomas, metastasizing to the colorectum. 90% of these cases were metachronous, with a preceding ovarian tumor. On gross examination, these were included predominantly in the bilateral ovarian tumors, with capsular breach and solid and cystic areas. On morphology, metastatic tumor deposits in the colorectum retained the morphology of ovarian papillary serous cystadeocarcinoma with foci of psammomatous calcification in 6/11 cases. Absence of 'garland-like' tumor necrosis, desmoplasia and LVI were pointers towards an ovarian primary, apart from the clinical context of a preceding ovarian tumor in 81.8% such cases. Wherever the colonic resection specimens were available, the tumor was predominantly submucosal or serosal, with absence of surface dysplasia, ruling out a colonic primary. In view of a definite clinical diagnosis with identical histomorphological features of the primary ovarian adenocarcinoma, IHC was restricted to 6 out of 11 such cases. In these cases the IHC pattern included CK7 and CA-125 positivity, with CEA and CK 20 negativity [16]. Four of these were metachronous (3 treated cases, post CT). Zighelboim et al [3] described a single case of atypical sigmoid metastasis from a high-grade ovarian adenocarcinoma, using differential expression of CK7 & 20. The plausible explanation of colorectal involvement in ovarian adenocarcinomas is through intraperitonel seedling. Hematogenous spread occurs in advanced peritoneal disease. In a substantial number of autopsy cases of advanced ovarian cancers, Rose et al [17] identified peritoneal involvement in 83–100% cases, with large intestinal involvement in 50–60% cases. An objective confirmation with specific cytokeratins in our 6/11 such unusual cases series has not been described earlier. In most of such cases, serosa was found to be initially affected and the remaining wall, thereafter. In their study, O' Hanlan et al [18] hypothesized that ovarian metastases to the gastrointestinal tract might mimic the colonic pattern of dissemination. In this setting, the invaded lymph channels at the submucosa of the bowel enter and replace mesenteric lymph nodes, forming large deposits of carcinoma in the paracolic and intermediate mesenteric groups. They concluded that a longitudinal negative margin of 2 – 5 cm in the resected bowel along with a wedge resection of mesentery, including paracolic and intermediate-level nodes might be indicated to achieve optimal debulking of gastrointestinal metastases from ovarian carcinomas.

Reardon et al [19] described an ovarian papillary serous cystadenocarcinoma mimicking recurrence at colorectal anastomosis in a previously diagnosed case of a colorectal adenocarcinoma, utilizing progesterone receptor (PR) expression. The possibility of double cancers in our study was ruled, based on the aforementioned clinicopathological features. Nonetheless there were challenging cases. A case of an ovarian tumor, on biopsy, showed an adenocarcinoma with both, 'garland-like' necrosis and psammomatous calcification. Presence of psammomatous calcification has been rarely documented in colorectal adenocarcinomas [20]. On IHC, diffuse CK20 and CEA positivity; with CK7 and CA-125 negativity reinforced a colonic primary that was further confirmed by presence of a rectal tumor. Another case presented with a rectal nodule, which was found to be a serosal deposit of a poorly differentiated adenocarcinoma. Imaging revealed an adnexal mass. Histopathologically, unremarkable rectal mucosa with CK7 positivity and CK 20 negativity in the tumor cells helped in ruling out a colonic primary. CK 7 is also known to be expressed in breast and the upper aerodigestive tract [9]. Additionally CA-125 positivity, along with elevated serum tumor markers levels of CA-125, confirmed an ovarian origin of the tumor.

## Conclusion

In cases of ovarian involvement by colorectal adenocarcinomas and vice versa, it can be difficult to ascertain an exact primary. In such cases, apart from complete clinical details, histomorphological features like 'garland-like' necrosis, desmoplasia; presence of lymphovascular emboli and psammomatous calcification are useful 'pointers' towards exact primary in this scenario. Differential expression of cytokeratins 7 & 20 by IHC is helpful in solving these dilemmas. CK7 negativity and CK20 positivity are indicative of a colonic adenocarcinoma. Additionally, frequent CEA positivity in colorectal tumor vs. CA-125 expression in ovarian tumors, improves the diagnostic accuracy. Metastatic tumors can present as unilateral ovarian tumors. More specific markers need to be further explored and validated with substantial studies to resolve these diagnostic dilemmas.

## Competing interests

The authors declare that they have no competing interests.

## Authors' contributions

BR designed the study and was involved in the diagnosis of some and review of cases; preparation and drafting of the manuscript with final version and the artwork. SG reviewed the cases, collected references, drafted and prepared the manuscript. BM assisted with designing and drafting of the manuscript. RD the Epidemiologist and biostatistician was involved in presentation and analysis of the data. RFC contributed to the overall supervision and gave the permission for the study. AM was the treating oncosurgeon and provided the cases with the clinical details. All authors have read and approved the manuscript.

## Supplementary Material

Additional file 1 Table 1. Clinicopathological features of 20 cases of ovarian involvement by colorectal adenocarcinoma and vice-versa. Click here for file

## References

[B1] Antila R, Jalkanen J, Heikenheimo O (2006). Comparison of secondary and primary ovarian malignancies reveals differences in their pre and perioperative characteristics. Gynaecol Oncol.

[B2] Singh N (2004). The pathology of metastases to the ovary. CME. Journal of Gynaecologic Oncology.

[B3] Zighelboim I, Broaddus R, Ramirez PT (2004). Atypical sigmoid metastasis from a high-grade mixed adenocarcinoma of the ovary. Gynaecol Oncol.

[B4] Sobrero AF, Aschele C, Bertino JR (1997). Fluorouracil in colorectal cancer – a tale of two drugs: implications for biochemical modulation. J Clin Oncol.

[B5] Lash RH, Hart WR (1987). Intestinal adenocarcinomas metastatic to the ovaries. A clinicopathologic evaluation of 22 cases. Am J Surg Pathol.

[B6] McCluggage WG, Wilkinson N (2005). Metastatic neoplasms involving the ovary: a review with an emphasis on morphological and immunohistochemical features. Histopathology.

[B7] Vang R, Gown A, Barry TS, Wheeler DT, Yemelyanova A, Seidman JD, Ronnett BM (2006). Cytokeratins 7 and 20 in primary and secondary mucinous tumors of the ovary: Analysis of coordinate immunohistochemical expression profiles and staining distribution in 179 cases. Am J Surg Pathol.

[B8] Mc Cluggage WG, Young RH (2005). Immunohistochemistry as a diagnostic aid in the evaluation of ovarian tumors. Semin Diagn Pathol.

[B9] Lagendijk JH, Mullink H, Van Diest PJ, Meijer GA, Meijer CJ (1998). Tracing the origin of adenocarcinomas with unknown primary using immunohistochemistry: Differential diagnosis between colonic and ovarian carcinomas as primary sites. Hum Pathol.

[B10] Campbell F, Herrington CS (2001). Application of cytokeratin 7 and 20 immunohistochemistry to diagnostic pathology. Curr Diagn Pathol.

[B11] Lewis MR, Deavers MT, Silva EG, Malpica A (2006). Ovarian involvement by metastatic adenocarcinoma. Still a diagnostic challenge. Am J Surg Pathol.

[B12] Daya D, Nazerali L, Farnk GL (1992). Metastatic ovarian carcinoma of large intestinal origin simulating primary ovarian carcinoma. A clinicopathologic study of 25 cases. Am J Clin Pathol.

[B13] Ramaekers F, van Niekerk C, Poels L, Schaafsma E, Huijsmans A, Robben H, Schaart G, Vooijs P (1990). Use of monoclonal antibodies to keratin 7 in the differential diagnosis of adenocarcinomas. Am J Pathol.

[B14] Moll R, Löwe A, Laufer J, Franke WW (1992). Cytokeratin 20 in human carcinomas. A new histodiagnostic marker detected by monoclonal antibodies. Am J Pathol.

[B15] Park SY, Kim HS, Hong EK, Kim WH (2002). Expression of cytokeratins 7 and 20 in primary carcinomas of the stomach and colorectum and their value in the differential diagnosis of metastatic carcinomas to the ovary. Hum Pathol.

[B16] Cathro H, Stoler MH (2002). Expression of Cytokeratins 7 and 20 in Ovarian Neoplasia. Am J Clin Pathol.

[B17] Rose PG, Piver MS, Tsukada Y, Lau TS (1989). Metastatic patterns in histologic variants of ovarian cancer. An autopsy study. Cancer.

[B18] O'Hanlan KA, Kargas S, Schreiber M, Burrs D, Mallipeddi P, Longacre T, Hendrickson M (1995). Ovarian carcinoma metastases to gastrointestinal tract appear to spread like colon carcinoma: implications for surgical resection. Gynecol Oncol.

[B19] Reardon CM, Kavanagh EG, Sabah M, Kirwan WO (1998). Ovarian cancer mimicking recurrence at colorectal metastasis. Dis Colon Rectum.

[B20] Nakayama H, Okumichi T, Nakashima S, Kimura S, Ikeda M, Kajihara H (1997). Papillary adenocarcinoma of the sigmoid Colon associated with psammoma Bodies and hyaline Globules: Report of a Case. Jap J Clin Oncol.

