# Supplementary material for: Clinicopathological features and the value of differential Cytokeratin 7 and 20 expression in resolving diagnostic dilemmas of ovarian involvement by colorectal adenocarcinoma and vice-versa
Source: Diagn Pathol. 2008 Sep 18;3:39. doi: 10.1186/1746-1596-3-39 (PMC2556647; doi:10.1186/1746-1596-3-39)
Supplement: Additional file 1 — Table 1. Clinicopathological features of 20 cases of ovarian involvement by colorectal adenocarcinoma and vice-versa. [file 1746-1596-3-39-S1.doc]

**Table 1** Clinicopathological features of 20 cases of ovarian involvement by colorectal adenocarcinoma and vice- versa.

| **Sr No.** | **Age** | **Presentation** | **Gross type** | **Laterality** | **Initial Diagnosis** | **CK7/CK20** | **CEA** | **CA125** | **Final Diagnosis** |
| --- | --- | --- | --- | --- | --- | --- | --- | --- | --- |
| 1 | 68 | S | So+C | B/L | Mucinous adeno. | +/++++ | + | NP | Mets from colorectum |
| 2 | 50 | S | NA | U/L | Mucinous adeno. | +/++++ | ++ | NP | Mets from colorectum |
| 3 | 57 | S | So | U/L | MD adeno. | NP | NP | NP | Mets from colorectum |
| 4 | 34 | M, Post CT | NA | U/L | MD adeno. (intestinal) | -/++++ | NP | NP | Mets from colorectum |
| 5 | 55 | S | So+C | B/L | MD adeno. | +/++++ | +++ | - | Mets from colorectum |
| 6 | 45 | M | NA | NA | PD adeno. ‘signet ring’ cell type | -/++++ | +++ | NP | Mets from colorectum |
| 7 | 58 | S | NA | NA | MD adeno. | -/++++ | +++ | - | Mets from colorectum |
| 8 | 68 | S | So+C | NA | MD adeno. | -/++++ | ++ | - | Mets from colorectum |
| 9 | 25 | M | So+C | U/L | MD adeno. | -/++++ | ++ | - | Mets from colorectum |
| 10 | 49 | M, 1^st^ ovary, Post CT | NA | B/L | PD endometroid adeno. | ++/- | - | ++++ | Mets from ovary |
| 11 | 23 | M, 1^st^ colon | So+C | B/L | Papillary serous cyst adeno. | ++++/- | NP | NP | Mets from ovary |
| 12 | 40 | M | So+C | B/L | Endometrioid adeno. | ++++/- | - | ++++ | Mets from ovary |
| 13 | 52 | M, Post CT | So+C | U/L | PD adeno. NOS | NP | NP | NP | Mets from ovary |
| 14 | 47 | M, Post CT | NA | NA | PD papillary serous cyst adeno. | +++/- | NP | NP | Mets from ovary |
| 15 | 63 | M | So+C | B/L | Papillary serous cyst adeno | NP | NP | NP | Mets from ovary |
| 16 | 51 | S | So | B/L | PD papillary serous cyst adeno. | NP | NP | NP | Mets from ovary |
| 17 | 49 | M, Post CT | NA | NA | PD papillary serous cyst adeno. | NP | NP | NP | Mets from ovary |
| 18 | 55 | M | NA | NA | PD adeno. NOS | +++/- | NP | - | Mets from ovary |
| 19 | 42 | S | NA | B/L | PD adeno. NOS | +/- | + | - | Mets from ovary |
| 20 | 34 | M | NA | B/L | Papillary serous cyst adeno | NP | NP | NP | Mets from ovary |

M: Metachronous. S: Synchronous. CT: Chemotherapy. So: Solid. C: Cystic. NA: Not Available. U/L: Unilateral. B/L: Bilateral. NA: Not available. PD: Poorly differentiated. MD: Moderately differentiated. Adeno: adenocarcinoma. NOS: Not other wise specified. NP: Not performed. Mets: Metastatic
